# Common oral complications of head and neck cancer radiation therapy: mucositis, infections, saliva change, fibrosis, sensory dysfunctions, dental caries, periodontal disease, and osteoradionecrosis

**DOI:** 10.1002/cam4.1221

**Published:** 2017-10-25

**Authors:** Herve Y Sroussi, Joel B. Epstein, Rene‐Jean Bensadoun, Deborah P. Saunders, Rajesh V. Lalla, Cesar A. Migliorati, Natalie Heaivilin, Zachary S. Zumsteg

**Affiliations:** ^1^ Division of Oral Medicine & Dentistry, Brigham and Women's Hospital Boston MA; ^2^ Samuel Oschin Comprehensive Cancer Instititue Cedars‐Sinai Medical Center Los Angeles CA; ^3^ Division of Otolaryngology and Head and Neck Surgery Duarte California; ^4^ Centre de Haute Energie 10, Bd Pasteur 06000 Nice France; ^5^ Department of Dental Oncology Health Sciences North Northeastern Cancer Centre Sudbury Ontario Canada; ^6^ Northern Ontario School of Medicine Rm 42036 Sudbury Ontario P3E 5J1 Canada; ^7^ Section of Oral Medicine University of Connecticut Health Farmington Connecticut; ^8^ Department of Oral and Maxillofacial Diagnostic Sciences University of Florida Gainesville Florida; ^9^ Oral Maxillofacial Surgery Department University of California San Francisco California; ^10^ Department of Radiation Oncology Cedars‐Sinai Medical Center Los Angeles California 90048

**Keywords:** Fibrosis, head and neck cancer, neurosensory disorder, oral candidiasis, oral health, oral mucositis, radiation therapy

## Abstract

Patients undergoing radiation therapy for the head and neck are susceptible to a significant and often abrupt deterioration in their oral health. The oral morbidities of radiation therapy include but are not limited to an increased susceptibility to dental caries and periodontal disease. They also include profound and often permanent functional and sensory changes involving the oral soft tissue. These changes range from oral mucositis experienced during and soon after treatment, mucosal opportunistic infections, neurosensory disorders, and tissue fibrosis. Many of the oral soft tissue changes following radiation therapy are difficult challenges to the patients and their caregivers and require life‐long strategies to alleviate their deleterious effect on basic life functions and on the quality of life. We discuss the presentation, prognosis, and management strategies of the dental structure and oral soft tissue morbidities resulting from the administration of therapeutic radiation in head and neck patient. A case for a collaborative and integrated multidisciplinary approach to the management of these patients is made, with specific recommendation to include knowledgeable and experienced oral health care professionals in the treatment team.

## Introduction

Cancer patients undergoing radiation therapy (RT) of the head and neck encounter acute and chronic changes to their soft tissue as well as transient and permanent sensory disturbances. In addition, RT results in a deterioration in dental and periodontal health as well as a risk of osteoradionecrosis. The acute effects of RT include mucositis, thickened secretions, mucosal infections, pain, and sensory disruptions. The long‐term chronic effects of head and neck RT comprise tissue fibrosis, salivary gland dysfunction, increased susceptibility to mucosal infections, neuropathic pain, sensory disorders and an increased susceptibility to dental caries and periodontal disease. The purpose of this article is to describe some of the common complications of head and neck radiation during and following cancer therapy and to discuss management strategies based on evidence and on the clinical experience of the authors.

### Mucositis

**Figure 1:**
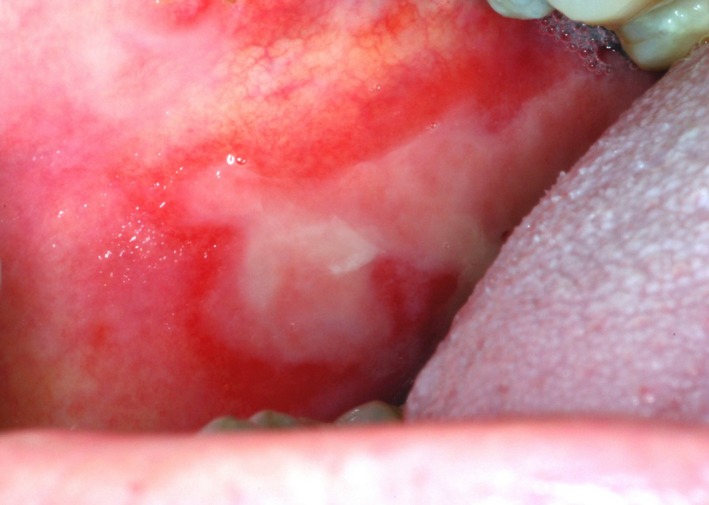
Oral Mucositis lesion on the buccal mucosa of a patient receiving radiation therapy to the head and neck region. Note the central area of ulceration covered by a whitish pseudomembrane, and the surrounding erythematous area. Picture from the teaching collection of Dr. Rajesh V. Lalla.

Oral mucositis (OM) is an acute response to treatment that affects the majority of the patients receiving RT for head and neck cancer (HNC) 1. In patients receiving a typical 6–7 week course of RT, OM presents as erythema of the oral mucosa in the first 2–3 weeks of RT and progresses to ulceration and pseudomembranes (Fig. 1) as the dose of radiation increases. Mucositis may be evaluated using mucositis scales such as the World Health Organization (WHO) mucositis scale, the National Cancer Institute (NCI) scale for oral mucositis and the Common Terminology Criteria for Adverse Events (CTCAE) (Table 1). The WHO mucositis scale is the most commonly used scale in clinical and research settings, whereas the NCI CTCAE scale is often used as a measure of overall toxicity. Other validated scales are available, and are primarily used in clinical research studies of mucositis (e.g.: OMAS Scale) 2. Although the anatomic distribution of mucositis is predominantly related to the radiation dose distribution, nonkeratinized oral tissues (buccal mucosa, lateral tongue, soft palate, floor of mouth) are more susceptible to OM than keratinized oral tissues 3. For HNC patients receiving concurrent chemotherapy and RT, OM may be more severe, appears earlier in the treatment course, and is of longer duration. Targeted therapies (e.g., epidermal growth factor inhibitors) amplify OM 4, and may cause dermatitis and resulting in extension to sites beyond the high‐dose RT fields.

**Table 1:** The World Health Organization Oral Mucositis Scale and The National Institute of Health Common Terminology Criteria for Adverse Events

Grade	Description
(A)	
0 (none)	None
I (mild)	Oral soreness, erythema
II (moderate)	Oral erythema, ulcers, solid diet is tolerated
III (severe)	Oral ulcers, only liquid diet is possible
IV (life‐threatening)	Oral alimentation is impossible
(B)	
0	None
1	Asymptomatic or mild symptoms; intervention not indicated
2	Moderate pain; not interfering with oral intake; modified diet indicated
3	Severe pain; interfering with oral intake
4	Life‐threatening consequences; urgent intervention indicated
5	Death

(A) Adapted from WHO Handbook 1979, pp. 15–22; (B) Adapted from NIH CTCAE v4.03 (2010) p. 45.

It is also important to assess patient reported outcomes (PROs), using validated scales such as: Vanderbilt Head and Neck Symptom Survey (VHNSS), Patient‐Reported Oral Mucositis Symptoms (PROMS), University of Washington‐QOL (UWQoL), Eastern Cooperative Oncology Group (ECOG), Functional Assessment of Cancer Therapy – Head and Neck (FACT‐HN) **[** 1, 5 **]**. The scale with broadest coverage of oral and head and neck symptoms is the VHNSS. The scale chosen is based upon the nature of the treatment provided and the potential goal of research and clinical care. The PROMs scale has been evaluated for use in settings where specific oral evaluation is not conducted and addresses mucositis specifically 6.

OM can peak near the end of RT and continue for 2 to 4 weeks post‐RT 7, 8, with recovery over several weeks, depending on the severity of the lesions and the addition of chemotherapy or targeted therapy. Diagnosis of OM is usually made clinically. However, secondary infection such as candidiasis or Herpes simplex virus (HSV) infection should be considered if the clinical appearance is unusual or duration is prolonged. The diagnosis of complicating infection can be challenging.

The primary morbidity of OM is pain associated with erythema and ulcerative lesions. Pain may lead to significant functional compromise affecting oral functions including nutrition. This may result in weight loss that may require use of a gastrostomy tube 9. Quality of life is significantly compromised by OM 1, 8. Severe OM may lead to emergency room visits, hospital admission and undesirable breaks in RT or discontinuation of planned chemotherapy. This may compromise the outcome of cancer therapy and increases the cost of care 9, 10.

Management of OM in HNC RT patients remains largely symptomatic. Many centers use a locally‐compounded mouth rinse (often referred to as “magic mouthwash”) containing lidocaine, often in combination with other ingredients such as diphenhydramine, a coating agent such as Maalox^®^, and occasionally an antifungal. However, mixing several active ingredients results in dilution of each agent and potential incompatibility of products that reduce their overall efficacy, and furthermore, there is limited clinical evidence that magic mouthwash is efficacious 11. In contrast, other agents, such as doxepin 12, palifermin 13, 14, benzydamine 15, and certain proprietary coating agents 16, have shown benefit in reducing mucositis pain in randomized controlled trials. Topical use of analgesics may be helpful and provide longer duration of pain relief; however systemic analgesics may be needed for pain management (see below). Evidence‐based clinical practice guidelines for OM have been published by the Multinational Association of Supportive Care in Cancer/International Society of Oral Oncology (MASCC/ISOO) 3. These guidelines include recommendations (based on higher level evidence), suggestions (based on lower level evidence), or a determination of “no guideline possible” (based on inadequate or conflicting evidence). Guidelines relevant to HNC RT‐induced OM are discussed below with the specific Level of Evidence (LOE) listed in brackets and can be found at http://www.mascc.org/mucositis-guidelines. MASCC/ISOO guidelines for oral mucositis and other toxicities of RT are also included in the “RadOnc Toolbox,” an app that will shortly be released by the Radiation Oncology Institute/ASTRO.

1. Oral Care: Standardized oral care protocols are recommended to prevent oral mucositis in all age groups and across all cancer treatment modalities (LOE III) 17.
2. Pain Control: It is suggested that 0.2% morphine mouthwash (LOE III) and 0.5% doxepin mouthwash (LOE IV) may be used for pain management due to OM in head and neck RT patients 17. No recommendation was possible for the use of combination mouthwashes (“magic mouthwash”) containing lidocaine and other ingredients, due to inadequate evidence.
3. Benzydamine: It is recommended that benzydamine mouthwash (anti‐inflammatory [not approved for mucositis in USA]) be used to prevent oral mucositis in patients with HNC receiving moderate dose RT (up to 50 Gy), without concomitant chemotherapy (LOE I). Benzydamine has not been adequately studied in RT with concomitant chemotherapy 12.
4. An additional study supports the use of benzydamine for patients receiving higher radiation dosage (≥50 Gy) with significant reduction in mucositis score starting at week 4 of the radiation treatment 15.
5. Low‐level laser therapy (LLLT) [photobiomodulation]: It is suggested that LLLT (wavelength 620–810 nm) be used to prevent OM in patients undergoing RT, without concomitant chemotherapy, for HNC (LOE III). No guideline was given for patients receiving concomitant chemotherapy, due to limited evidence 18. The mechanisms may include anti‐inflammatory and analgesic effects as well as promotion of healing 19, 20.
6. Zinc: It is suggested that systemic oral zinc supplements may be of benefit for preventing OM in oral cancer patients receiving RT or chemoradiation (LOE III). The mechanism may include promotion of wound healing 21.

The MASCC/ISOO guidelines also provide guidelines against the use of agents shown to be ineffective. For HNC RT patients, the use of sucralfate mouthwash (coating agent), topical antimicrobials (polymyxin, tobramycin, amphotericin B; bacitracin, clotrimazole, gentamicin), chlorhexidine mouthwash (antimicrobial), misoprostol mouthwash (anti‐inflammatory), and systemic pilocarpine (saliva stimulant) was not recommended 13, 22, 23. It should be noted that while these agents are not recommended for OM, some of these agents may have application for other indications in this population.

### Oropharyngeal candidiasis

**Figure 2:**
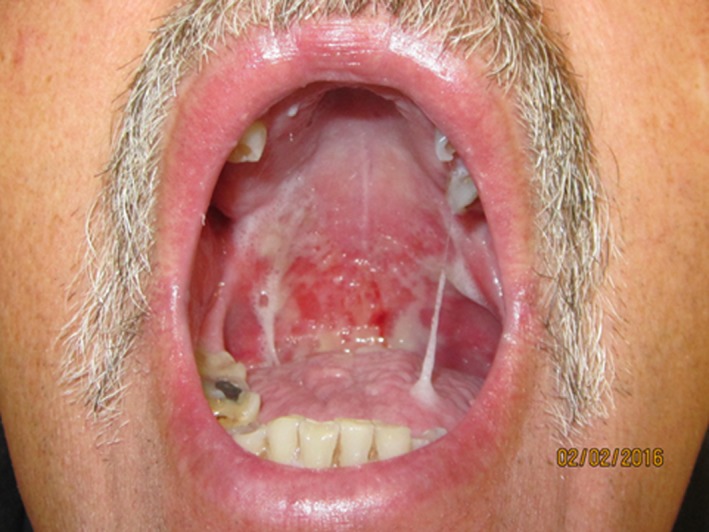
Head and neck cancer patient at 25/35 fraction with dry ropey saliva, oral mucositis, and suspected oral candidiasis. Picture from Dr. Deborah Saunders.

Oropharyngeal candidiasis (OPC) is common in HNC patients (Fig. 2). OPC is associated with mucosal pain, taste change and can extend to the esophagus and result in dysphagia; in addition to oropharyngeal symptoms, oral intake can be adversely affected affecting nutritional status and ability to take oral medications. Regional extension or systemic dissemination may occur in myelo/immunosuppressed patients. Clinical presentation includes pseudo‐membranous (thrush) and erythematous candidiasis, and angular cheilitis. Hyperplastic (nodular) and invasive candidiasis are less common and may require biopsy for diagnosis.

Candidiasis has variable symptoms: from no symptoms to burning sensitivity and pain, a sensation of coating in the mouth, odynophagia, dysgeusia (often described as a metallic taste), and smell of yeast infection. Diagnosis can be challenging, because the symptoms may overlap with those of mucositis. In some cases, differentiation between candidiasis and mucositis can be made by the presence of angular cheilitis, erythema, and pseudomembranes outside of the high‐dose radiation volume. The differential diagnosis of fungal glossitis includes geographic tongue, lichen planus, erythema multiforme, herpetic lesions, leukoplakia, oral hypersensitivity, denture reaction, and hairy leukoplakia. Microbiological study is not diagnostic of infection as, the presence of noninvasive yeast in the oral cavity may simply represent a carriage state frequently seen in healthy people 24. Organism identification may be needed in resistant infection and to confirm the clinical diagnosis. It is not uncommon to evaluate the response to antifungal treatment as a diagnostic strategy to rule out oral candidiasis.

Current guidelines for management of OPC derive primarily from clinical trials in immunosuppressed HIV patients 25. Topical oral treatments are recommended as first‐line therapy in milder forms of candidiasis 26. Topical azole or polyene antibiotics in the form of a lozenge, suspension or cream may be applied intra‐orally. Instructions include applying nystatin and amphotericin B (available in Europe) 27 four to six times daily, maintaining contact time on the mucosa as long as possible. It should be noted that the use of amphotericin B lozenges did not perform as well as systemic fluconazole in this comparative study 27. Some products, including oral rinse forms of nystatin that contain sugar, leads to increased caries risk particularly in dentate patients with hyposalivation 28, 29. Topical fluconazole rinses can be compounded and have been examined and shown effective in cancer patients with candidiasis. Topical clotrimazole is available in a lozenge and cream but the assumption that it use as a topical agent completely avoids concerns of systemic exposure and drug‐drug interactions is not supported by reports of such complications 30. Topical miconazole is available in cream form, and in a muco‐adhesive tablet (Loramyc^®^ EU; Oravig^®^ USA) that does not have sugar sweetening used once daily and has a broad spectrum of activity against Candida species 26.

Systemic treatments should be used in case of failure of local treatment or immediately with severe clinical OPC in high risk (myelosuppressed, immunocompromised) patients. In general, systemic therapy with fluconazole (Triflucan^®^, Diflucan^®^) is superior to topical antifungals in cancer patients 31. Among the systemically used azoles, fluconazole appears to have the fewest drug interactions. The Infectious Diseases Society of America recommends 200 mg on day one (loading dose) followed by 100 mg/day for OPC 27. Fluconazole can be used for prophylaxis in cases with frequent recurrences using 50–200 mg/day or 100–400 mg/week, and has been shown superior to oral polyenes 32. Nicolatou‐Galitis et al. demonstrated a significant reduction in candida carriage and an elimination of oral candidiasis together with a significant reduction in severe mucositis and treatment interruption at the end of RT in subjects randomized to a group receiving a daily dose of 100 mg fluconazole when compared to controls^33^. The effectiveness of this approach has yet to be confirmed by others and it is not widely implemented. If candidiasis develops during RT, antifungal therapy should continue until completion of planned RT and patients should also be followed after completion of RT to determine if candidiasis recurs in order resume therapy or institute preventive protocols. However, the widespread use of fluconazole has been associated with the emergence of fluconazole resistant fungae 34. Voriconazole and posaconazole have demonstrated efficacy in esophageal candidiasis in immunocompromised patients but is not recommended for use for treatment of initial or mild cases of OPC. A Cochrane review of management of candidiasis in cancer patients receiving radiation and/or chemotherapy did not identify sufficient evidence to support the use of current interventions in treating oral candidiasis recommending that additional studies be conducted to address this gap of knowledge 35.

The use of chlorhexidine is not recommended for management of mucositis, but may have value as a broad spectrum antiseptic to control microbial risk of dental and gingival disease and has limited antifungal activity 36. If used in patients with oral mucositis formulations without alcohol are needed.

### Neurosensory disorders: mucosal pain and taste dysfunction

Mucosal pain that affects diet and quality of life occurs during active cancer therapy and is a common chronic complaint in survivors 37. Mucosal pain may be related to inflammation, ulceration, mucosal atrophy or mucosal neuropathy and may be compounded by dry mouth and potentially secondary infection. Radiation‐induced neuropathies may be caused by neurotoxicity, ischemia, oxidative stress, and inflammation 38. The onset of neuropathic symptoms is variable beginning during cancer therapy or may be delayed in onset. Mucosal sensitivity may persist long after clinical mucositis resolves and is common at one‐year follow‐up and reported in up to two‐thirds of patients 39. A number of chemotherapeutic agents used in HNC may lead to neuropathy, including platinum agents, taxanes, fluorouracil and targeted agents. 40. Targeted chemotherapy may cause mucosal ulceration (aphthous‐like) and neurotoxicity resulting in pain which may represent the treatment limiting toxicity 41. Immunotherapies (immune check point inhibitors) may stimulate immune/inflammatory processes leading to pain in the oral mucosa and oropharynx 42.

Pain prevention and management will improve with further understanding of the molecular and neurophysiologic mechanisms underlying the painful condition (Tables 2 and 3) 43, 44. Current management follows guidelines of WHO analgesic ladder with reduced emphasis on opioid analgesics, which have limited effect on neuropathic pain, and a focus on adjunctive centrally‐acting medications and pain management strategies (Table 4). Such factors as serotonin, norepinephrine, substance P, calcitonin gene related peptide, N‐methyl D‐aspartate, prostaglandins, COX‐2, tumor necrosis factor‐alpha, vascular endothelial growth factor, nerve growth factor, altered tissue pH, interleukins and nociceptor sensitization and stimulation may represent potential targets of therapy.

**Table 2:** Mechanisms of Mucosal pain in oncology

Processes		
Radiation therapy	Acute	Mucositis, infection, molecular sensitization and stimulation
	Chronic	Neuropathy, atrophy, hyposalivation, ischemia, fibrosis; molecular sensitization and stimulation
Chemotherapy/targeted therapy, immunotherapy	Acute	Mucositis; infection, molecular sensitization and stimulation
	Chronic	Neuropathy, fibrosis; molecular sensitization and stimulation

**Table 3:** Potential molecular sensitizers and mediators of pain

Neurotoxicity/neuropathy
Radiation, chemotherapy, targeted therapies
Cellular necrosis and apoptosis:
Cell contents ↑ inflammation, nociception
Tumor acidity, inflammation ↓ pH, proton induced pain
Inflammatory mediators: damaged tissue and inflammation
Cytokines/growth factors:
Tumor Necrosis factor (TNF), interleukins (IL‐1, IL‐6), Platelet derived growth factor (PDGF), epidermal growth factor (EGF), transforming growth factor (TGF), vasoactive intestinal peptide (VIP), vascular endothelial growth factor (VEGF), nerve growth factor (NGF), endothelins, others
Sensory Neurotransmitters:
Serotonin, noradrenaline, bradykinin, substance P, Calcitonin gene related peptide (CGRP), excitatory amino acids (e.g., glutamate;activation N‐methyl‐D‐aspartate receptors),protons, reactive oxygen species
Inflammatory mediators:
Proinflammatory cytokines, histamine
Arachadonic acid metabolites: prostaglandins, leukotrienes, adenosine, adenosine 5'‐triphosphate, nitric oxide
Other:
Infection:
Microbial waste products, pH, increase inflammation, proinflammatory cytokines, inflammatory cell activity

**Table 4:** Management of oral mucosal pain

Symptom management:
Topical agents: anesthetics, analgesics, neurologically active medications
WHO ladder:
Analgesics (prostaglandins, COX2)
Nonsteroidal analgesics, acetominophen
Mild opioid combination agents
Strong opioids and nonsteroidal analgesics, acetominophen
Adjunctive Centrally acting medications:
Anticonvulsants
Antidepressants
Tricyclics, gabapentinoids, serotonin norepinephrine reuptake inhibitors
Anxiolytics; sleep promoters
Adjunctive techniques:
Acupuncture, low‐level laser therapy (LLLT), psychological techniques
Psychological:
Cognitive/behavioral therapy
Distraction techniques
Relaxation/ imagery techniques
Music therapy; drama therapy
Counseling

There is no proven prophylaxis for mucosal pain, although it is anticipated that less severe mucositis and reduced duration of ulceration may lead to reduced risk of long‐term mucosal sensitivity. Current management of persisting neuropathic pain associated with or following mucositis relies upon neurologically active medications including clonazepam, gabapentin, pregabalin, duloxetine, and tricyclics. Topical delivery may be considered when local sensitivity is seen. Other approaches include acupuncture and low‐level laser therapy (photobiomodulation) 45, 46.

HNC RT and chemotherapy may directly modify smell and taste sensations 47. RT may cause neuroepithelial damage and chemotherapy and targeted therapy may also result in a dysgeusia or ageusia and mucosal sensitivity 48. Taste disorders occur in more than 75% of HNC patients receiving RT 49. The recovery of taste following RT is variable. In some cases improvement is noted within 2–6 months after treatment, but in some, the changes may continue ad infinitum 49, 50, 51, 52. Flavor is a combination of taste, smell, texture, and temperature. Flavor impacts food choices, food intake, and therefore influences nutritional status. Taste is a complex sensation, based upon five basic qualities (sweet, bitter, salt, sour, and umami [savory]). In addition, fat “taste,” spicy “sensation” and metallic taste may play a role mediated by receptor transduction, nonspecific transport across the cell membrane 51 and may stimulate a chemosensory response which may enhance flavor and be important in energy intake. Small C‐fiber function mediates sensations that are a component of taste including capsaicin (hot‐spicy sensation), piperine (pungency of black pepper), and zingerzone (perception of ginger), as well as sensations induced by menthol (cooling sensation) 51. Change in C‐fiber and A‐delta fibers in mucosal sensitivity may impact taste.

Taste and flavor have had limited study in cancer therapy. In addition to RT, chemotherapy and targeted therapeutics may affect taste by direct taste receptor stimulation, or damage, or via secretion in saliva or gingival crevicular fluid 52. Hyposalivation and oral, dental, periodontal, and oropharyngeal pathologies may affect taste function and mucosal sensitivity 53, 54 It should be recognized that systemic factors can influence taste such as paraneoplastic syndromes 55, diabetes, severe anemia, and leukemia 56. In addition, medications may have taste‐related side effects, with the highest prevalence rates of taste change associated with antibiotics, anti‐hypertensives, antidepressants, muscle relaxants, and multiple cancer chemotherapeutics including cytotoxic, targeted and immune therapies 57.

All taste qualities are affected with RT to the oral cavity. Initially, sweet perception may decrease, resulting in symptoms of increased bitter and salt taste, followed by general abnormal taste and reduction in taste perception 58, 59. Umami taste may decrease during RT and recovery of umami taste may be delayed and continue indefinitely. Loss of umami taste is important because it may reduce interest in eating and negatively impact quality of life 51. Damage to C‐fibers may result in mucosal sensitivity and change in taste.

Management includes dietary counseling with guidance in food choices, food preparation and seasoning (increase spice if tolerated, increase umami foods, and umami flavoring), and avoiding unpleasant foods. Zinc sulfate supplementation has been therapeutically tested with inconsistent outcomes in clinical studies 60, 61. Centrally acting medications have received limited study to date, but early suggestions include: clonazepam, gabapentin, cannabinoids, and megestrol 61.

### Post radiation fibrosis

Late effects from HNC RT may involve several different structures in the regions that were irradiated. In particular, radiation to the neck may cause damage to the vessels, nerves, and muscles. Moreover, damage to the lymphatics, which drain fluid from the head and neck, may cause lymphedema. Fibrosis in lingual muscles as well as constrictor muscles of the pharynx can follow therapy and may affect tongue function and swallowing, respectively. Fibrosis in masticatory muscles, particularly lateral pterygoids can result in trismus.

Trismus, temporomandibular disease, and fibrosis limiting function of the lips and tongue may develop as late RT side effects. RT to the muscles of mastication (masseter, temporalis, and the medial and lateral pterygoids) and region of the temporomandibular joint 62 causes inflammatory changes which can lead to muscle fibrosis 63. Trismus can lead to difficulty in eating, swallowing, speech, general oral hygiene and access for use of dental prosthesis and delivery of dental care.

The incidence of radiotherapy‐induced trismus (RTIT)in HNC patients varies greatly in older studies, ranging between 5% and 45% which may be largely attributed to a lack of uniform criteria for RTIT and changes in RT technique 64. A cut‐off point of <35 mm for mouth‐opening was introduced in 2006 and has been used as a standard for trismus in latest studies 65. The incidence of trismus is decreasing with intensity‐modulated radiotherapy (IMRT) for most HNC patients receiving curative radiation treatment. Similarly, pharyngeal constrictor muscle damage has decreased with the last generation of IMRT machines. A systematic review of 12 articles from 1990 to 2008 66 reported the weighted prevalence for RTIT to be 25% in patients receiving three‐dimensional conventional radiotherapy (3D‐CRT), 5% for IMRT, and 31% in combined CRT and 3D‐CRT. The authors argued that the effects of RT are cumulative with an initially slow onset and that RTIT may begin soon after the end of RT or at any time up to 2 years posttreatment with variable progression remain the same or improve over time 66.

Although RTIT has been documented in the literature for decades, research on prevention and treatment is limited. Two systematic reviews from 2004 and 2010, one researching the effect of different cancer treatments on the prevalence of trismus 64, and the other attempting to identify criteria, risk factors and treatment interventions for trismus 66, concluded that there is a need for appropriately powered, prospective randomized studies to better understand and manage RTIT.

Pentoxifylline and vitamin E may reduce treatment related fibrosis, but the level of evidence was not sufficient for guideline use 66. Use of a device developed for trismus (TheraBite* Jaw Motion Rehabilitation System) was suggested due to low level evidence in favor of use.

The efficacy of Low Level Light Therapy (LLLT) or photobiomodulation has potential application for prevention and treatment of RTIT and is now evaluated in several clinical studies 67. While international evidence‐based guidelines for patients with HNC experiencing RTIT are lacking 67, clinical regional guidelines have been developed, suggesting that RTIT may be prevented by active, passive and supportive stretching of the muscles of mastication during RT.

### Dental caries

Patients are at increased risk of dental caries following RT primarily due to hyposalivation 68. Saliva has essential functions in maintaining tooth structure due to control of pH, remineralization and antimicrobial and tooth cleansing effects 69. Dental caries results from a loss of equilibrium in tooth demineralization‐remineralization culminating in mineral loss leading to damage to the organic phase of tooth structures, resulting in cavitation. Demineralization first appears as increased white lesions involving gum line regions and cusp tips of the teeth. Untreated caries can progress rapidly and require more extensive treatment making prevention and early detection of mineral loss essential. Sequelae include pain, infection of the jaw bone, and potentially the need for tooth extraction and in patients who have high‐dose RT to the region, resulting in risk of osteoradionecrosis.

The prevalence of caries in patients who have received RT depends on the cohort, but averages well above 25% 70. The risk of caries in cancer survivors has been shown to correlate with the dose of RT to the parotid glands, 71, 72 and it is expected that parotid sparing regimen will reduce the risks of caries. Hey et al. 72 reported that patients who did not experience new carious lesions 24 months after RT received a statistically lower RT dose (21.2 ± 11.4 Gy) compared to patients with sporadic (26.5± 11.59 Gy) or generalized caries lesions (33.9 ± 9.93 Gy). Increase in the incidence of carious lesions correlated with decline in stimulated whole saliva secretion. While data on submandibular gland exposure is limited, it is anticipated that function of these glands are also important in maintaining dental health. Irradiation of the salivary glands results in hyposalivation, and causes changes in the composition of saliva 73, decreasing its ability to prevent dental demineralization.

The term “radiation caries” 74 has been used to describe rampant caries following HN RT. Although radiation can directly affect the structure and mechanical properties of teeth 75, 76, there is little evidence that the pathogenesis of radiation caries is different from that of the classical caries in other patient populations with hyposalivation. In addition, radiation caries appear clinically similar to nonradiation related caries seen in dry mouth patients 76. However, radiation associated caries develop more rapidly and are more likely to include nonclassical surfaces of teeth (cusp tips, gum line cavities) when compared to classical caries. Radiation caries are also associated with a higher rate of recurrence and a greater risk of failure of the dental treatment requiring additional dental procedures 77. The increased risk is related to demineralization, shift to a more cariogenic oral flora, difficulty in oral hygiene and possible shift to a diet high in carbohydrates 78.

In our experience, it is recommended that patients who have or will undergo RT maintain an aggressive comprehensive oral health management plan. This includes regular dental care which will allow early identification of demineralization and carious lesions and fluoride and calcium applications to support dental remineralization. While fluoride delivered in application trays to the teeth is considered the most effective means of application, different methods of fluoride application are available and comparative effectiveness for fluoride application methods is not documented. The application of fluoride can be accomplished in professionally applied fluoride varnishes, with mouth washes, high fluoride prescription toothpaste, complex fluoride slow‐release devices 79, 80. The latter technique may be cumbersome and expensive without necessarily yielding better results than simple fluoride trays. Fluoride applications must be continued as long as hyposalivation persists. If the patient is not compliant with the use of fluoride carriers, brush on fluoride on teeth twice daily and other cavity prevention (i.e., diet modifications) must be undertaken.

Early detection of caries through dental follow up visits every 6 months are recommended to preserve oral health. More frequent follow‐ups may be necessary depending on persistence of hyposalivation and the presence/progression of dental demineralization, caries, and periodontal status.

In patients with documented demineralization of teeth, and if saliva production is reduced, use of remineralizing products (calcium and phosphate) are necessary to provide building blocks of teeth in addition to topical fluoride 81. The use of antiseptic chlorhexidine to reduce cariogenic microbial load 82 and sialagogues 83, 84 to stimulate salivary flow should also be considered. Stimulation of salivary production may be achieved in patients with residual saliva production using sugar‐free or alcohol‐substituted sugar (e.g., xylitol) lozenges, which may be supplemented with fluoride or other elements for caries prevention 85. Dietary and oral hygiene counseling 83 is a critical part of the management of patients before, during, and after RT of the head and neck. The inclusion of the proactive dental management plan described above, together with an effort to spare salivary function and to stimulate residual gland function, can improve caries control 86 in high‐risk oncology patients. This requires the awareness of the oncology team of the patient's oral health needs as well as the training of dentists in delivering care to the oncology patient, as general dentists may feel ill equipped and reluctant to treat these patients 87.

### Periodontitis

Periodontitis is a highly prevalent and chronic microbial/inflammatory disease which is characterized by the loss of tooth‐supporting tissue inclusive of the tooth supporting alveolar bone. Periodontitis may culminate in pain, infection of the jaw bones around dental roots, and tooth loss. In HNC patients, periodontitis may be a trigger of osteoradionecrosis (ORN) (see below) 88. Patients receiving radiation for head and neck cancers are at an increased risk for periodontal disease compared to the general population for several reasons. Hyposalivation and the loss of the protective effects of saliva may predispose to periodontitis. Furthermore, the use of RT in the head and neck region causes changes in the oral microbiome, with a shift to periodontal disease‐associated flora 78, 89. Consistently, rapid loss of tooth‐supporting tissue was noted by Ammajan et al. 90 who reported a significant loss in periodontal attachment level as well as gingival recession when comparing patients pre‐ and post‐RT.

The effect of RT on periodontal health is dose‐dependent and is associated with worsened periodontal health following the initiation of RT 91. Independent of the risk of tooth‐loss, periodontal disease is relevant to the management of the oncology patients as it has been linked to an increased risk of ORN 88 and also to oral mucositis (OM) 92, 93. Pre‐existing periodontitis, which is common in adults, is likely to worsen with cancer treatment. Furthermore, progressive periodontitis may lead to the need for tooth extraction which may itself result in ORN. It should also be noted that early malignant lesions can mimic periodontitis 94, 95 and expert evaluation by an oral health professional may facilitate a proper diagnosis.

Current recommendations state that patients be examined and treated by a dentist who is aware of the planned cancer therapy and of oral issues before, during, and following cancer therapy 82. Periodontal disease is the primary cause of tooth loss in adults and extractions of teeth with severely compromised periodontium may be required before the initiation of oncology treatment ‐ particularly in a region of planned high‐dose RT. Teeth that have periodontal attachment loss and teeth that are anticipated to require surgical management in the future within the planned high‐dose RT fraction should be extracted prior to RT. Teeth involved may have mobility, or periodontal pockets that require full dental examination to be identified. A lifelong commitment to preventative oral health management is required to minimize the risk of worsening periodontitis which could require a tooth extraction with the associated risks of ORN (discussed immediately below).

### Oral complications of head and neck radiotherapy in elderly patients

Elderly patients represent a unique subset of patients in whom acute and late adverse sequelae of RT can be particularly challenging. Increasing age is associated with increased risk of severe late toxicity, such as dysphagia, aspiration pneumonia, and long‐term feeding tube dependence on prospective RTOG protocols 96. However, there is limited prospective data specifically comparing oral toxicity in elderly patients undergoing RT to what is observed in younger patients. Furthermore, there is no consensus on the definition of elderly with some defining it as 70 years or older while others use a 65‐years cut‐off 97, 98, 99. Most comparative data comes from retrospective case series, with conflicting conclusions. In a study of patients enrolled on EORTC trials from 1980 to 1995, those 65 years of age and older had higher grade 3–4 mucositis during radiation than younger patients, but there was no difference in late toxicities including trismus, xerostomia, or dysphagia 97. However, other series have reported no difference in acute oral complications for elderly and younger patients undergoing radiotherapy 67, 68. Additionally, higher hospitalization rates for elderly patients and unplanned treatment breaks have been reported in some series, 98, 100, 101 but this appears more commonly due to infectious or renal complications rather than oral complications. In contrast, some major centers have not seen increased hospitalizations or treatment breaks in elderly patients 102. Thus, further investigation into the impact of age and comorbidity on radiation complications is warranted.

### Osteoradionecrosis

Osteoradionecrosis (ORN) is the result of ischemic necrosis of the bones associated with soft tissue necrosis without the presence of tumor 53. Histopathological findings include a pre‐fibrotic phase with increased endothelial cells activity and inflammation, a subsequent phase characterized by abnormal fibroblastic activity, and a final phase with characteristic fibroatrophic remodeling and loss of osteocytes in bone 54. Histopathological findings include: initially hyperemia, endarteritis and thrombosis, followed by cell loss, hypovascularity, increase in bone marrow fat and fibrosis 55. The histomorphometric analysis was statistically significant for hypocellularity, hypovascularity and fibrosis 55.

Reports have indicated a variable incidence of ORN between 4 and 37% 56 with declining risk in modern series associated with advances in RT. When three‐dimensional RT and IMRT became available, the observed average incidence of ORN decreased to lower than 5% 57. In a systematic review, the weighted prevalence of ORN was 7.4% in conventional RT, 5.1% with IMRT, 6.8% with chemoradiation (CRT), and 5.3% with brachytherapy 58. In addition, the lower incidence of ORN has also been attributed to improved preventive oral care 59. When oral hygiene is poor and other local factors are present, such as ill‐fitting dentures or post‐RT dentoalveolar surgery, the incidence can exceed 25% 60.

ORN has been classified based on treatment response to Hyperbaric Oxygen Therapy (HBO) 61; on the clinical behavior of ORN whether healed, chronic but nonprogressive, or active progressive 62; or the severity grade according to the anatomical extent 63. Other classifications include the extent of ORN and symptoms 64.

ORN may cause significant loss of quality of life particularly in advanced stages 65. Risk factors associated with ORN include tumor‐related factors, treatment‐related factors, and patient‐related factors 55. Some of these factors include the presence of dental disease (inflammation and infection), the need for pre‐irradiation HNC surgery and dental surgery, oral health and hygiene 53. Others factors predicting ORN include increased RT fraction size, increased total RT dose to the mandible, and other factors such as the extent of the tumor, proximity with bone, bone invasion and the need for pre‐radiation bone resection 66. Males age 55 or older with history of tobacco and alcohol use are more frequently affected 56, 67. The mandible is more commonly affected than the maxilla. While most ORN cases develop in the first three years after completion of RT 53, 63, 68, it can occur at any time following RT.

Treating existing oral disease and stabilizing oral health before and following cancer therapy may decrease the risk of ORN. The goal is to minimize the need of invasive interventions (e.g., extraction) and dental inflammatory disease/infection during and after RT for the life of the patient. Evaluation by an experienced dental provider and integration of care with the oncology team will determine ideal oral care prior to starting radiation therapy.

Management protocols proposed to treat ORN include conservative therapy with medication, ultrasound, HBO, and surgical resection and reconstruction for nonresponding, advanced stage ORN 53. The characterization of fibrosis as part of the pathogenesis of ORN has led to the use of anti‐radiation fibrosis drugs such as pentoxifylline, tocopherol, and clodronate 69. Antioxidant therapy has also been proposed 70. New experimental studies have assessed bone marrow‐derived stem cells and bone morphogenetic protein‐2 to facilitate osseous healing in cases of ORN 71. Other approaches include promotion of bone and soft tissue repair using low‐level laser therapy 72. Resection and vascular graft reconstruction are considered in cases with pathologic fracture or threatening fracture with progression despite nonsurgical therapy. The best approach to treating ORN is prevention. Additional prospective studies are needed to determine long‐term success of ORN management. There is little evidence other than clinical case series to support the use of these treatment modalities including the use of HBO 73. A controlled trial of HBO for the management of mandibular ORN showed it to be without merit 74.

## Conclusion

This paper outlines some of the key dental, oral soft tissue, and neurological complications in HNC treated with RT. Oral care must include the full trajectory of the cancer journey from diagnosis to survivorship. Preventing, assessing, and managing oral complications throughout the active continuum of care are required to promote the best possible patient QOL. The pretreatment dental management should be directed at necessary assessment including complete oral, dental, and periodontal examination and baseline range of jaw movement and saliva production. The baseline measures allow evaluation of changes that may occur following treatment and indicate potential need for intervention. Standard preventive oral care should be provided. Acute oral complications may be more easily recognized than the ongoing issues common in survivors, but both represent a challenge to the oncology team. Integrated pretreatment oral and dental evaluation of HNC patients is the best approach to achieving the best possible clinical outcomes. It is also clear that a life‐long access to expert oral health care is critical for HNC survivors, as loss of motility and sensory disturbances may remain permanent.

One of the key challenges in achieving optimal integrated oral health care for HNC patients is that not all cancer centers have integrated oral and dental care programs. Some centers obtain services from general hospital dentistry programs, often delivered by one‐year general practice residents, with limited supervision. Some of the HNC patients are seen by community oral health providers, the majority of which have no training or experience in oncology and are not integrated with the oncology team and may not be prepared to treat the dental/oral needs 103. Recommendations for identifying community resources that may assist the cancer center in obtaining oral care have been discussed elsewhere 104.

## Conflict of Interest

The authors disclose no conflict of interest.
